# Immuno-metabolic profile of human macrophages after *Leishmania* and *Trypanosoma cruzi* infection

**DOI:** 10.1371/journal.pone.0225588

**Published:** 2019-12-16

**Authors:** Maureen C. Ty, P’ng Loke, Jordi Alberola, Ana Rodriguez, Alheli Rodriguez-Cortes

**Affiliations:** 1 New York University School of Medicine, Department of Microbiology, New York, NY, United States of America; 2 Dept Farmacologia, Toxicologia i Terapeutica, Facultat de Veterinaria, Edifici V, Universitat Autonoma de Barcelona, Bellaterra, Spain; Louisiana State University, UNITED STATES

## Abstract

Macrophages can reprogram their metabolism in response to the surrounding stimuli, which affects their capacity to kill intracellular pathogens. We have investigated the metabolic and immune status of human macrophages after infection with the intracellular trypanosomatid parasites *Leishmania donovani*, *L*. *amazonensis* and *T*. *cruzi* and their capacity to respond to a classical polarizing stimulus (LPS and IFN-γ). We found that macrophages infected with *Leishmania* preferentially upregulate oxidative phosphorylation, which could be contributed by both host cell and parasite, while *T*. *cruzi* infection did not significantly increase glycolysis or oxidative phosphorylation. *Leishmania* and *T*. *cruzi* infect macrophages without triggering a strong inflammatory cytokine response, but infection does not prevent a potent response to LPS and IFN-γ. Infection appears to prime macrophages, since the cytokine response to activation with LPS and IFN-γ is more intense in infected macrophages compared to uninfected ones. Metabolic polarization in macrophages can influence infection and immune evasion of these parasites since preventing macrophage cytokine responses would help parasites to establish a persistent infection. However, macrophages remain responsive to classical inflammatory stimuli and could still trigger inflammatory cytokine secretion by macrophages.

## Introduction

Macrophages are versatile cells in the immune system that play an essential role in fighting infections. Besides the most characteristic tasks as phagocytic killers and antigen-presenting cells, macrophages are essential in the control of the inflammatory response and in the repair of damaged tissues. Macrophages can express different functional programs in response to environmental signals to adapt their function to the needs of a particular immunological situation. Typically, macrophages can be polarized to classically activated (M1) or alternatively activated (M2) subsets in response to the surrounding stimuli. M1 macrophages are characterized by a high microbicidal capacity through reactive oxygen and nitrogen species and secretion of pro-inflammatory cytokines, while M2 macrophages produce high levels of anti-inflammatory cytokines and express cell surface markers that participate in tissue remodeling and resolution of inflammation [1].

The process of macrophage polarization is modulated by metabolic reprogramming [2]. The metabolism of activated immune cells does not only cover their biosynthetic and bioenergetic demands, but also acts as a switch to control their immune functions [3]. M1 metabolism mainly relies on glycolysis and pentose phosphate pathway [4–6]. Glycolytic enzymes support the inflammatory response, such as the enzymes pyruvate kinase 2 and glyceraldehyde-3-P-dehydrogenase inducing IL-1β and TNF, respectively [7, 8]. M1 macrophage metabolism is also characterized by a truncated TCA cycle, leading to increased succinate and citrate levels which drive the generation of IL-1β and NO, respectively [6, 9]. On the other hand, M2 macrophages present a metabolism based in glucose oxidative phosphorylation, oxidation of fatty acids, and increased utilization of glutamine to support their functions of long term wound healing and defense against extracellular parasites [10–12]. Editing macrophage polarization is emerging as a new therapeutic approach for cancer and atherosclerosis [13].

Pathogens that infect macrophages have sophisticated adaptations to thrive in a host cell that is specialized in the elimination of microbes. Two trypanosomatid parasites, *Leishmania* and *Trypanosoma cruzi*, that replicate successfully in macrophages, use different mechanisms to evade killing in the phagosome. *Leishmania* is protected from the oxidative attack in the phagolysosome by a thick cover layer of lysophosphoglycan and is also able to interfere with the generation of reactive oxygen and nitrogen radicals [14]. *T*. *cruzi* evades the phagosomal attack by disrupting the internalization vacuole and escaping free in the cytosol [15].

Here, we infected monocyte-derived human macrophages with *Leishmania* or *T*. *cruzi* to analyze the effect of infection on the metabolic activation and polarization of macrophages. We found that while *Leishmania* infection of macrophages resulted preferentially in increased oxidative phosphorylation over glycolysis, *T*. *cruzi* infection did not significantly increase glycolysis or oxidative phosphorylation. Neither infection induced the secretion of inflammatory cytokines by macrophages, which are typical of the M1 phenotype. However, glycolysis and inflammatory cytokines could be induced by activation of infected macrophages with IFN-γ and LPS, indicating that parasite infection did not inhibit M1 polarization and suggesting that during infection, macrophages are still able to respond effectively to an external activation stimulus.

## Methods

### Generation of human monocyte-derived macrophages

Whole blood was isolated from healthy donors at the Clinical and Translation Institute (CTSI) of NYU School of Medicine. This study (number 09-1536) was approved by the NYU School of Medicine Institutional Review Board (IRB) and was performed according to the guidelines established by the IRB. Written consent was obtained for all participants. Peripheral venous blood was kept in constant agitation with 7.22% Sodium citrate (Sigma) during the blood draw. PBMCs were isolated by density gradient centrifugation utilizing 30% of Ficoll-Paque PLUS (GE Life Sciences), and allowed to attach on treated tissue culture dish (Falcon). After 2 hours, plates were washed twice leaving only strongly adherent monocytes. After an overnight incubation, isolated monocytes were plated in six XF24 cell culture microplates (Agilent Technologies) and three regular 24-well culture plate (Corning) at a concentration of 250,000 cells/well in DMEM (Corning) and 1% Penicillin, Streptomycin and Glutamine mixture (PSG). Cells were allowed to differentiate for 7–12 days in 10% Human Sera (Valley Biomedical), when two XF24 plates and one regular plate were infected with each of the three parasites. After 18 hours, the two XF24 cell culture microplates were used for Seahorse analysis and the one regular 24-well culture plate (with glass coverslips in each well) was used for quantification of infection and determination of cytokines and chemokines in the supernatant. Infections were quantified by microscopy after fixation with methanol and staining of coverslips with Giemsa.

### *T*. *cruzi* culture

*T*. *cruzi* parasites from Brazil strain expressing the firefly Luciferase gene [16] were cultivated in 3T3 fibroblasts in DMEM with 2% FBS and 1% PSG. Trypomastigote forms were released in the culture medium of infected 3T3 cells and harvested 4 days after infection. The harvested medium was spun for 7 min at 1200 *g*, rinsed twice and pelleted. In order to eliminate the amastigotes, the trypomastigotes were allowed to swim out of the pellet for 3 h, after which supernatants containing *T*. *cruzi* were harvested, counted and used to infect macrophages (1 macrophage: 5 trypomastigotes).

### *L*. *amazonensis* and *L*. *donovani* culture

*L*. *donovani* and *L*. *amazonensis* (LV78) [17] were cultured in RPMI with 10% FBS 1% PSG kept at 26°C. At 6 days of culture, when *Leishmania* promastigotes reach their stationary phase, parasites were spun at 1800 *g* for 10 minutes, re-suspended and used to infect macrophages (1 macrophage: 10 parasites).

### LPS and IFN-γ stimulation

6 h after infection macrophages were stimulated or not with LPS (1 μg/ml) and IFN-γ (1000U/ml).

### Seahorse

Bioenergetic profile of infected macrophages was measured by determining oxygen consumption rate (OCR) and extracellular acidification rate (ECAR) in a XF-24 Flux Analyzer (Seahorse Bioscience). Sensor cartridges (Agilent Technologies) were hydrated in XF Calibrant (Agilent Technologies) at 37°C overnight following manufacturer’s instructions. Media for Glycolysis stress assay: XF Base Medium (Seahorse Bioscience) with 2mM glutamine, 1mM pyruvate, 10mM glucose, 5mM HEPES and MitoStress assay: XF Base Medium with 2mM glutamine, 1mM pyruvate and 10mM glucose were made fresh and kept at pH 7.4. Experimental plates were kept at 37°C without CO_2_ for 1 hour before loading into the Seahorse machine. Cells were sequentially treated with 10 mM glucose, 1μM oligomycin (O) and 50 mM 2-deoxy-glucose (2DG) to determine glycolysis parameters from the ECAR levels, or with 1 μM O, 1 μM FCCP (carbonyl cyanide-4-(trifluoromethoxy) phenylhydrazone), and 0.5 μM rotenone (ROT) plus antimycin A (AA) to asses OXPHOX parameters from the OCR levels.

### Cytokine and chemokine measurements

Media from cultures of infected macrophages was collected at 18 hours post-infection and stored at -80°C. Cytometric Bead Array (CBA, BD biosciences) was used to determine levels of cytokines (IL-8, IL-10, IL-1β, IL-6 and TNF) and chemokines (CXCL5, CXCL9, CCL2 and CXCL10). Cytokine and bead mixtures were performed on the FACs Calibur (BD biosciences) according manufacture’s protocol. FCAP array (BD Biosciences) was used for quantification.

### Viability determination with PrestoBlue

After Seahorse analysis of macrophages, wells were washed twice with PBS, and PrestoBlue Cell Viability Reagent (Invitrogen) diluted 1:10 with medium was added. Samples were then incubated for 3 hours at 37°C before measurement of absorbance at 595 nm.

## Results

We have investigated the metabolic status of human macrophages infected with the intracellular trypanosomatid parasites *Leishmania* and *T*. *cruzi*. Human macrophages were differentiated *in vitro* from monocytes isolated from healthy donors and infected with *Trypanosoma cruzi* trypomastigotes or with promastigotes from two different species of *Leishmania*: *L*. *donovani and L*. *amazonensis* which cause visceral and mucocutaneous leishmaniasis, respectively.

We observed that 18 h after addition of the human-infective stages of these parasites, macrophages were efficiently infected with *L*. *amazonensis* (average 85% of macrophages infected; 5.1 amastigotes/macrophage), *L*. *donovani* (average 86.7% of macrophages infected; 5.3 amastigotes/macrophage) or *T*. *cruzi* (Brazil strain; average 79.6% of macrophages infected; 3.5 amastigotes/macrophage). Viability of host cells assessed by PrestoBlue was 100% for the three infections. At this time, a Seahorse extracellular flux analyzer was used to study two major pathways of cellular energy production: glycolysis as measured by extracellular acidification rate (ECAR) and oxidative metabolism based on mitochondrial oxygen consumption rate (OCR).

**Fig 1:**
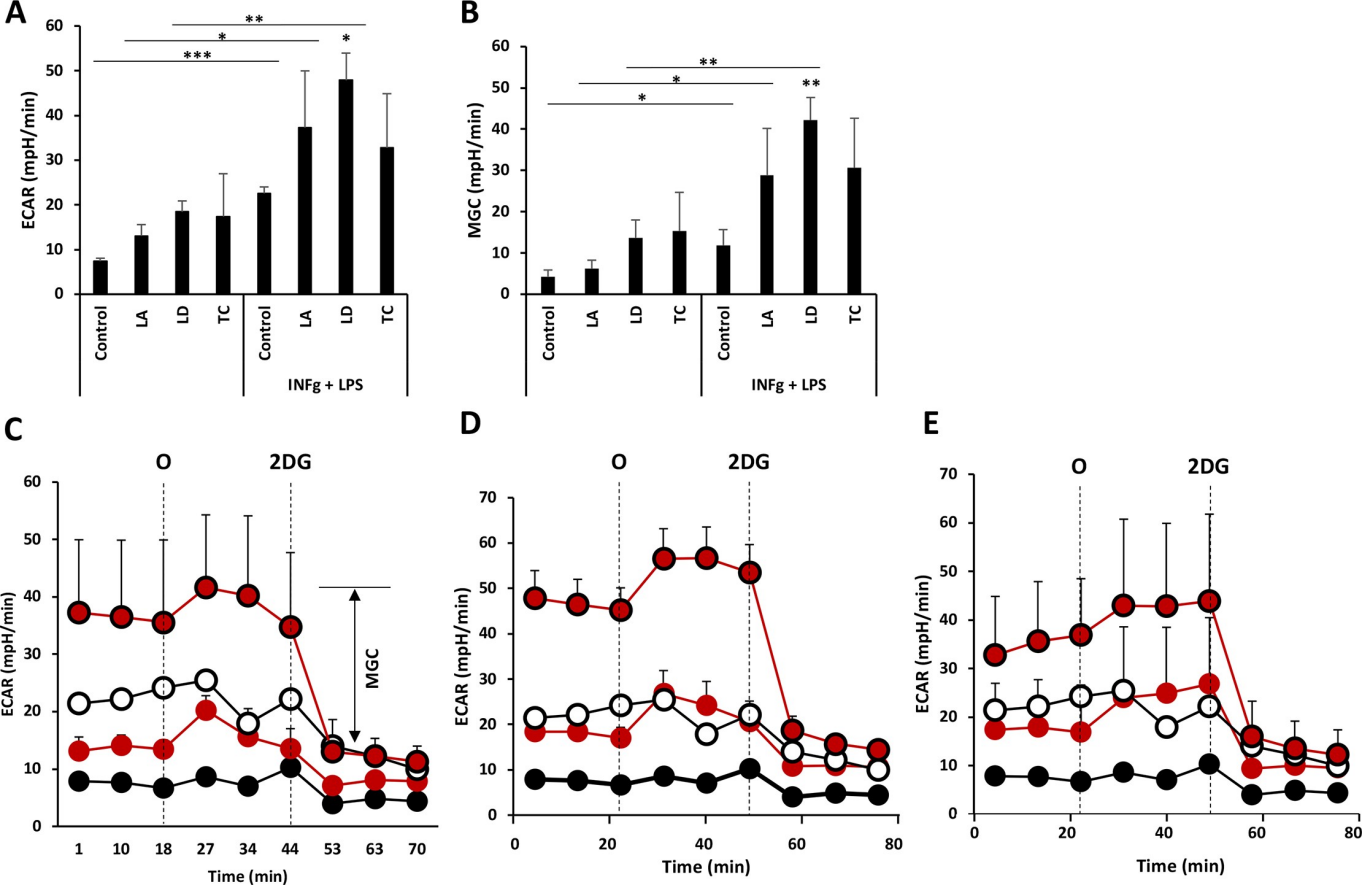
*L*. *amazonensis*, *L*. *donovani* and *T*. *cruzi* infection do not increase basal or maximal glycolytic capacity of macrophages. Glycolytic capacity in the presence of glucose as measured by ECAR (A) and maximal glycolytic capacity (MGC) (B) of macrophages infected with the indicated parasites for 18 h and treated or not with IFN-γ+LPS 6 h before starting Seahorse determinations. (C-E) Real-time changes in ECAR of macrophages infected with *L*. *amazonensis* (C), *L*. *donovani* (D) or *T*. *cruzi* (E) after treatment with oligomycin (O) (1 μM) and 2-deoxy-glucose (2DG) (50 mM). Parasite-infected macrophages are shown in red circles and IFN-γ+LPS stimulation in bold edges. MGC (double-headed arrow), is shown in (C). Average and standard deviation of triplicates from one experiment are shown. *p<0.05, **p<0.01 and ***p<0.001 by one-way ANOVA compared with control or IFN-γ+LPS control. Stars on lines indicate ANOVA comparison of IFN-γ+LPS treated cells to unstimulated ones. Independent stars indicate ANOVA comparison of cells infected with each parasite to control uninfected cells. One representative experiment out of 2 is shown.

To analyze how glycolysis is affected by *Leishmania* and *T*. *cruzi* infection in resting or activated macrophages, we recorded the extracellular acidification rate before and after addition of OM (an ATP synthase inhibitor), followed by 2DG, which inhibits glycolysis and allows correction for non-glycolytic acidification. We observed that infection with *L*. *amazonensis*, *L*. *donovani* or *T*. *cruzi* does not significantly increase glycolysis or maximal glycolytic capacity in macrophages compared to uninfected control macrophages (Fig 1). When uninfected control or *Leishmania*-infected macrophages were activated with IFN-γ and LPS 6 h after infection, we observed higher levels of glycolysis and of maximal glycolytic capacity (Fig 1). The increase in glycolysis indicates that infection did not inhibit the capacity of macrophages to respond to the activation stimuli. Although not statistically significant, a similar profile was observed for *T*. *cruzi*-infected macrophages.

**Fig 2:**
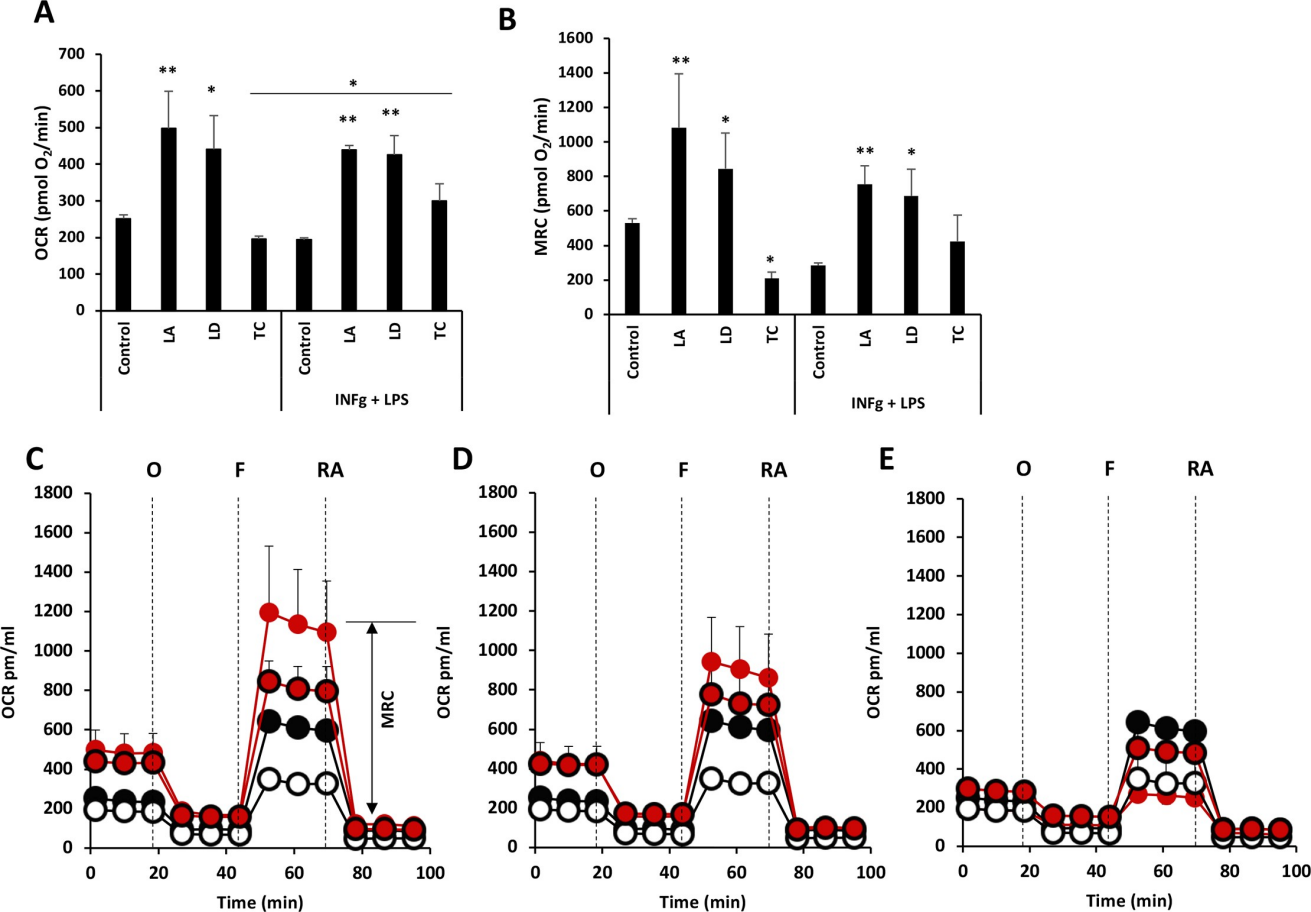
*L*. *amazonensis* and *L*. *donovani*, but not *T*. *cruzi* infection, increase basal and maximal respiratory capacity of human macrophages. Basal (A) and maximal (B) respiratory capacity of macrophages infected with the indicated parasites for 18 h and treated with or not with IFN-γ+LPS 6 h before the starting Seahorse determinations. (C-E) Real-time changes in OCR of macrophages infected with *L*. *amazonensis* (C), *L*. *donovani* (D) or *T*. *cruzi* (E) after treatment with oligomycin (O) (1μM), FCCP (F) (1μM), and rotenone and antimycin A (RA) (0.5μM). Parasite-infected macrophages are shown in red circles and IFN-γ+LPS stimulation in bold edges. Maximal respiratory capacity (MRC; double-headed arrow) is shown in (C). Average and standard deviation of triplicates from one experiment is shown. *p<0.05, **p<0.01 and ***p<0.001 by one-way ANOVA compared with control or IFN-γ+LPS control. Lines indicate ANOVA comparison of IFN-γ+LPS treated cells to unstimulated ones. Independent stars indicate ANOVA comparison of cells infected with each parasite to control uninfected cells. One representative experiment out of 2 is shown.

To determine the functional metabolic profile of mitochondria, we measured real-time changes in OCR during sequential treatment of cells with oligomycin, FCCP (a H^+^ ionophore) and a combination of rotenone and antimycin A (inhibitors of the electron-transport chain). We observed that both basal and maximal respiratory capacity are increased by infection with both *Leishmania* species, *L*. *amazonenesis* and *L*. *donovani*, when compared with uninfected macrophages. On the contrary, infection with *T*. *cruzi* resulted in no significant changes in basal OCR and a decrease of maximal respiratory capacity was detected (Fig 2B).

When control macrophages were incubated with IFN-γ and LPS we did not observe an increase in mitochondrial respiration, which is compatible with the M1-like phenotype induced by these stimuli [18]. Macrophages infected with *L*. *amazonenesis* or *L*. *donovani* did not modify the already increased level of basal and maximal mitochondrial respiration, while *T*. *cruzi* minimally increased this metabolic pathway (Fig 2).

Taken together these results indicate that leishmanial infection induces increased oxidative phosphorylation in macrophages, which could be a result of both increased mitochondrial respiration in the macrophage and oxidative phosphorylation performed by intracellular Leishmania. Post-infection treatment with IFN-γ and LPS resulted in increased glycolysis. On the other hand, *T*. *cruzi* infection does not induce changes in basal glycolysis or mitochondrial respiration and only minimally increases respiration upon addition of IFN-γ and LPS.

**Fig 3:**
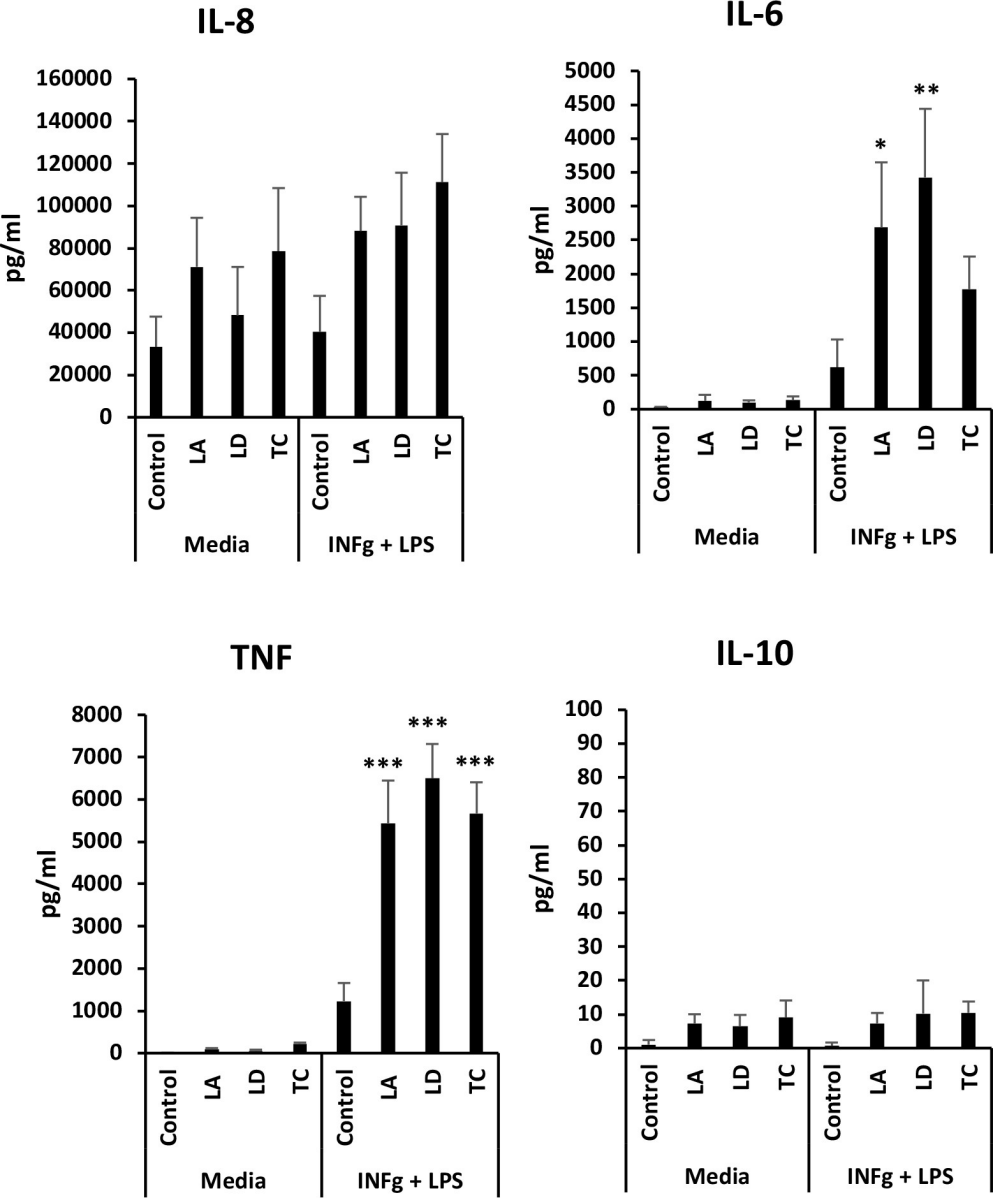
Infection of macrophages with *L*. *amazonensis*, *L*. *donovani* or *T*. *cruzi* does not induce inflammatory cytokine secretion by resting macrophages. Culture media of macrophages infected with *L*. *amazonensis* (LA), *L*. *donovani* (LD) or *T*. *cruzi* (TC) for 18 h and treated with IFN-γ+LPS or not 6 h before was used for inflammatory cytokine determinations. Average and standard deviation of triplicates from one experiment is shown. *p<0.05, **p<0.01 and ***p<0.001 by one-way ANOVA compared with control or IFN-γ+LPS control. One representative experiment out of 2 is shown.

Analysis of cytokine production by macrophages showed that infection with *L*. *amazonensis*, *L*. *donovani* or *T*. *cruzi* only induces very low intensity secretion of inflammatory cytokines such as IL-6, IL-8, and TNF (Fig 3), while other cytokines where undetectable (IL-2, IL-4, IL-5 and IFN-γ, indicating that macrophages do not mount an effective inflammatory response to infection with these parasites. However, inflammatory cytokines in infected macrophages were induced in response to IFN-γ and LPS (Fig 3), indicating that the capacity of macrophages to secrete cytokines was not inhibited. Indeed, macrophages infected with any of the three parasites secreted higher levels of TNF in response to activation compared with uninfected macrophages, showing that the increase in glycolytic metabolic potential is matched by increased cytokine secretion when infected macrophages are activated.

**Fig 4:**
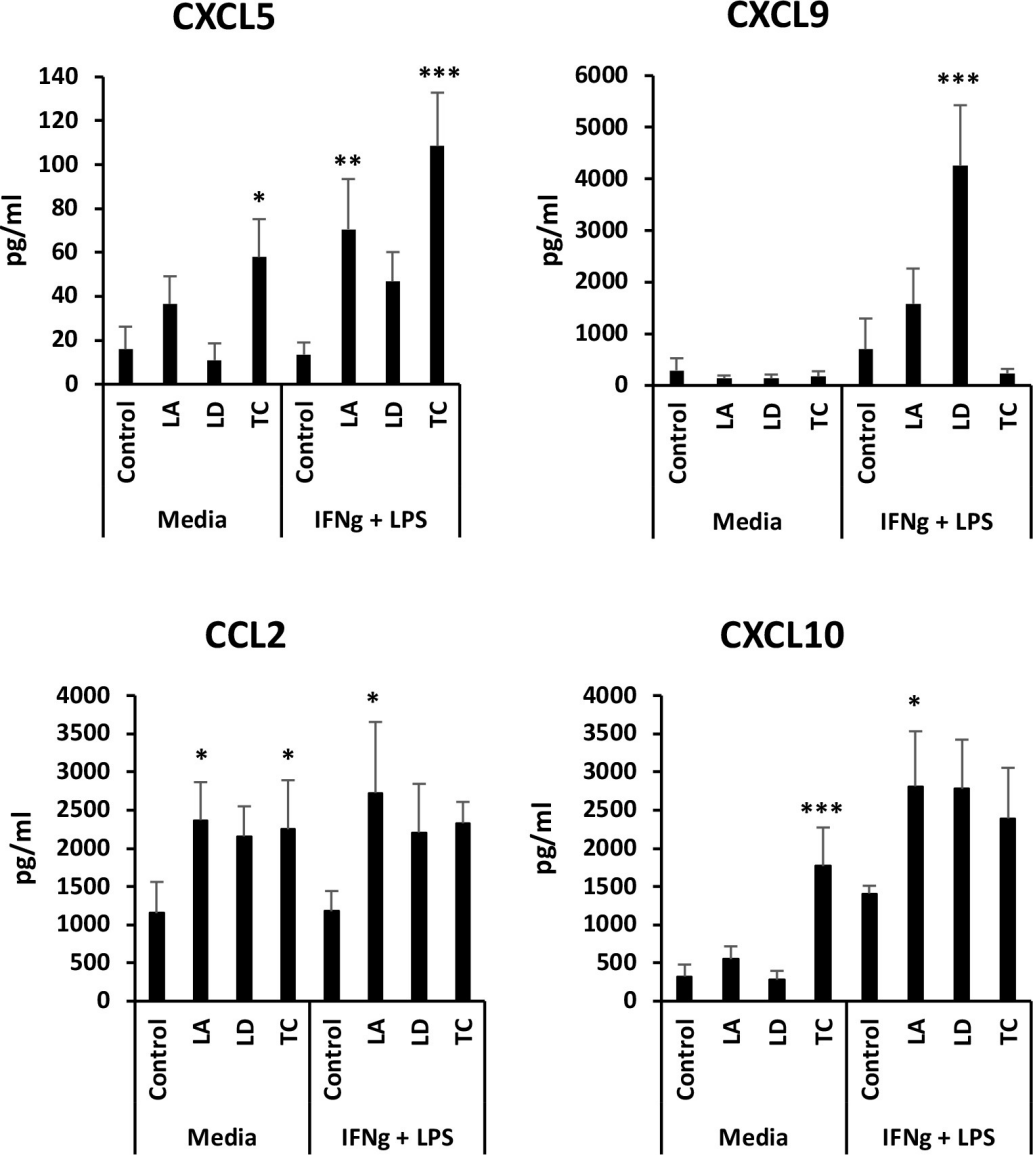
Infection of macrophages with *L*. *amazonensis*, *L*. *donovani* or *T*. *cruzi* induces secretion of specific chemokines by resting macrophages. Culture media of macrophages infected with *L*. *amazonensis* (LA), *L*. *donovani* (LD) or *T*. *cruzi* (TC) for 18 h and treated with IFN-γ+LPS or not 6 h before was used for chemokine determinations. Average and standard deviation of triplicates from one experiment is shown. *p<0.05, **p<0.01 and ***p<0.001 by one-way ANOVA compared with control or IFN-γ+LPS control. One representative experiment out of 2 is shown.

Analysis of a panel of chemokines showed that *Leishmania* infection induce little to no chemokine increases, although activation with IFN-γ and LPS produced a greater response when compared to uninfected macrophages (Fig 4), similarly to what observed with IL-6 and TNF (Fig 3). *T*. *cruzi* infection induced significantly the secretion of CCL2, CXCL5 and CXCL10, but not CXCL9 (Fig 4). Activation of *T*. *cruzi*-infected macrophages with IFN-γ and LPS induced increases only of CXCL5 (Fig 4).

## Discussion

Macrophage polarization in response to single-molecule stimuli, such as LPS or IL-4, has been extensively studied and has set the basis for the characterization of macrophages into M1 or M2 metabolic programs [5]. During host infection by pathogens, macrophages must encounter at the same time microbes carrying multiple effector molecules and environmental factors including cytokines and other immune mediators that would result in a variety of phenotypes ranging from strict M1 to M2 [19].

Another source of complexity is provided by the specific host-pathogen interactions that are established between each microbe and its host. Recent evidence has uncovered a connection between cellular effector functions and specific metabolic pathways, that is especially relevant in immune cells such as macrophages, since it can determine pathogen elimination or survival. However, the outcome of this interaction appears to be microbe-specific, since infection-induced glycolysis limits *Mycobacterium tuberculosis* survival [20], but increases *Brucella abortus* growth in macrophages [21].

Macrophage polarization in *T*. *cruzi* and *Leishmania* infections has been recently reviewed [22, 23] but the link between macrophage immunoprofile and cellular metabolism during these parasitic infections is still unclear. Our study was designed to analyze the effect of the intracellular trypanosomatid parasites *Leishmania* and *T*. *cruzi* infection on macrophage polarization from an immuno-metabolic perspective. We have used primary cultures of human monocyte-derived macrophages from healthy donors incubated with whole infectious parasites *in vitro*, in an attempt to represent at least part of the complexity of a real infection.

We observed that after 18 h of infection with *T*. *cruzi*, glycolysis was not significantly increased in macrophages. Similarly, a recent report that analyzed the metabolic profile of a macrophage cell line infected with a different strain of *T*. *cruzi* found a similar level of glycolytic metabolism in infected and uninfected macrophages [24]. We also observed minimal inflammatory cytokine secretion, in agreement with previous gene expression studies which revealed that genes induced by *T*. *cruzi* during early infection of macrophages are limited to type I interferon-stimulated genes, with no activation of classical pathways for inflammatory cytokines [25]. However, the inflammatory response of macrophages to *T*. *cruzi* is complex and varies greatly with time, since for example an inflammatory cytokine like TNF is upregulated 3 h after addition of *T*. *cruzi*, but downregulated at 18h [26]. It is possible that additional factors like parasite strain and/or macrophage origin could also affect the response. Our determinations were performed using primary human monocyte-derived macrophages in an attempt to better model human infections.

We observed secretion of several chemokines in response to *T*. *cruzi* infection (CCL2, CXCL5 and CXCL10), which indicate that despite the lack of inflammatory cytokines, the macrophages are not completely immunologically unresponsive to infection. These chemokines would induce the recruitment of monocytes, dendritic cells and T cells to the site of infection initiating innate and adaptive immune responses [27]. In mice *T*. *cruzi* infections, CXCL9 and CXCL10 were found to be expressed by macrophages and to contribute to parasite clearance [28].

Addition of activation stimuli (LPS + IFN-γ) to *T*. *cruzi*-infected macrophages did not result in a significant increase in glycolysis, suggesting that the infected cells are not fully responsive to activation, a characteristic that may shape the immune response during infection. Indeed, inflammatory cytokines, except for TNF, were not increased upon LPS + IFN-γ stimulation.

*T*. *cruzi* infection did not increase the oxidative metabolism of macrophages and it even induced a decrease in the maximal respiratory capacity of macrophages. A previous report had found also a small decrease in Seahorse-detected oxidative metabolism in a *T*. *cruzi*-infected mouse macrophage cell line that was further decreased when IFN-γ was added [24]. Since intracellular *T*. *cruzi* amastigotes growth is favored when cellular metabolism is fueled by lipid peroxidation [29], the low oxidative metabolism in macrophages may help controlling parasite growth in this cell type. However, metabolic responses appear to be different in non-phagocytic cells, where increased mitochondrial respiration was observed after *T*. *cruzi*-infection of fibroblasts and myoblasts. In this case, both parasite and host cell contributed to the increased respiration [30]. Overall, these results indicate that *T*. *cruzi* infection is intricately related to the metabolic status of the host cell and has the capacity to modulate it.

Infection of macrophages with *L*. *amazonensis* or *L*. *donovani* resulted in the activation of oxidative phosphorylation, a pattern that is also found in M2 polarized macrophages [10] and that is also compatible with the lack of inflammatory cytokine secretion by these macrophages [31]. The differential contribution of the host macrophages versus intracellular Leishmania in the increased oxidative phosphorylation has not been determined in our experiments, however previous studies in mouse macrophages infected with *L*. *infantum* showed a shift from glycolysis to oxidative phosphorylation that was driven by manipulation of host AMPK pathway [32], suggesting an important contribution of host macrophages to the measured increase in oxidative respiration. Additionally, several studies of gene expression profiles in *L*. *mexicana* and *L*. *donovani* infected macrophages presented a M2-like activation profile [25] [33], which is typically associated with increased oxidative respiration [3].

Infection of *Leishmania* infection is characterized by a silent phase [34] with no production of cytokines and a general down regulation of macrophage gene expression [35]. Since oxidative phosphorylation is not associated with inflammatory cytokine secretion in human macrophages, and AMPK activation reduces cytokine production [36] it is likely that *Leishmania* infection may suppress host inflammatory response by macrophage metabolic manipulation.

Previous reports had described that while M1 polarized macrophages fail to repolarize to M2 phenotype due to mitochondrial disfunction, IL4-induced M2 macrophages are highly plastic and can be reprogrammed into M1-like inflammatory state [37]. In our experiments, addition of activation stimuli (LPS + IFN-γ) to *L*. *donovani* infected macrophages resulted in a significant increase in glycolysis, indicating that the infected cells are still responsive to activation, a very important characteristic during infection. They produced an even higher secretion of inflammatory cytokines compared to uninfected macrophages, indicating that infected macrophages increased metabolic capacity was matched by the increased cytokine production in response to the stimuli. These results confirm the capacity of parasite-infected macrophages with minimal inflammatory cytokine secretion to be activated to a M1-like profile (increased glycolysis and inflammatory cytokine secretion).

Our results indicate that *Leishmania* and *T*. *cruzi* infect macrophages without triggering a strong inflammatory cytokine response, but infection appears to ‘prime’ macrophages, since the cytokine response to activation with LPS and IFN-γ is more intense in infected macrophages compared to uninfected ones. These parasites would have the capacity to evade innate immunity by preventing macrophage cytokine responses during infection, which would be accompanied by a metabolic manipulation of the infected macrophage. This study opens new possibilities for the study of macrophage metabolic rewiring as a new therapeutic strategy.
